# Non rigid respiratory motion correction in whole body PET/MR imaging

**DOI:** 10.1186/2197-7364-1-S1-A73

**Published:** 2014-07-29

**Authors:** Hadi Fayad, Holger Schmidt, Christian Wuerslin, Dimitris Visvikis

**Affiliations:** INSERM UMR1101, LaTIM, Brest, France; Université de Bretagne Occidentale, Brest, France; University Hospital of Tübingen, Kragujevac, Germany

Respiratory motion in PET/MR imaging leads to reduced quantitative and qualitative image accuracy. Correction methodologies include the use of respiratory synchronized gated frames which lead to low signal to noise ratio (SNR) given that each frame contains only part of the count available throughout an average PET acquisition. In this work, 4D MRI extracted elastic transformations were applied to list-mode data either inside the image reconstruction or to the reconstructed respiratory synchronized images to obtain respiration corrected PET images.

Five patient datasets acquired on the SIEMENS mMR PET/MR system were used. T1-weighted 4D MR images were registered to the end expiration MR image using an elastic B-spline registration algorithm to derive deformation matrices used to account for the respiratory motion. The derived matrices were subsequently applied during the List Mode OSEM PET image reconstruction of the original emission list-mode data. The corrected images were compared with those produced by applying the elastic transformation after reconstructing PET frames followed by summing the realigned gated frames, as well as with uncorrected motion averaged images.

Significant improvement was obtained by using any of the two respiratory motion correction techniques compared to uncorrected motion average images (>30% differences in tumor position and size and 25% SNR increase). Both correction approaches lead to nearly equivalent improvements (differences of <8% and <6% for the lesion FWHM and the SNR respectively).

A list-mode reconstruction based respiratory motion correction for PET has been implemented and its performance evaluated. This approach was based on the use of elastic transformations derived from 4D MRI during PET image reconstrucion. Our results show significant respiratory motion compensation when compared to the motion average PET images, with slightly better contrast and SNR compared to an equivalent 4D PET image space elastic motion correction method.

